# A FoxO–Autophagy–Lipid Mobilization Axis Regulates Fat Body Remodeling During Honeybee Metamorphosis

**DOI:** 10.3390/insects17070684

**Published:** 2026-07-01

**Authors:** Jing Yu, Hongfang Wang, Zhenguo Liu, Ying Wang, Baohua Xu

**Affiliations:** 1 College of Sports and Leisure, Shandong Sport University, Jinan 250102, China; 2 College of Animal Science and Technology, Shandong Agricultural University, Taian 271000, China

**Keywords:** *Apis mellifera*, autophagy, fat body, FoxO, lipolysis

## Abstract

Forkhead box O (FoxO) transcription factors act downstream of insulin signaling and play conserved roles in development and metabolic homeostasis in insects. However, whether FoxO participates in 20-hydroxyecdysone (20E)-mediated pupation and fat body remodeling in honeybee larvae remains unclear. Here, we show that FoxO is highly expressed during the prepupal and pupal stages of honeybee development. RNA interference (RNAi)-mediated silencing of FoxO delayed pupation, inhibited ecdysteroid biosynthesis and 20E signaling, and ultimately led to pupal lethality. Knockdown of FoxO also suppressed the expression of lipolytic genes, reduced lipase activity, and increased triglyceride (TG) accumulation in the fat body. Furthermore, FoxO deficiency impaired autophagy, as evidenced by reduced LysoTracker staining, decreased autophagosome formation, and downregulation of *Atg* genes. These findings demonstrate that FoxO participates in 20E-induced pupation of honeybee by regulating the expression of key genes involved in 20E biosynthesis and the 20E signaling pathway. FoxO coordinates autophagy and lipid mobilization in the fat body to provide energy for pupal development. Collectively, our results establish FoxO as a central regulator linking endocrine signaling, energy metabolism, and tissue remodeling during honeybee metamorphosis.

## Simple Summary

Honeybees transform from larvae to adults through a pupal stage. This process needs precise hormone control and a lot of energy. We investigated whether a protein called FoxO is involved. When we reduced FoxO activity in larvae, they could not pupate properly and most died before becoming pupae. We found that FoxO helps a molting hormone (ecdysone) to work correctly. FoxO also helps the larval “fat body” (similar to the human liver) break down fats and clean out old cell parts through autophagy, providing energy for metamorphosis. Our work shows how FoxO connects hormone signals and energy metabolism to ensure successful transformation into adult honeybees.

## 1. Introduction

Members of the Forkhead box O (FoxO) transcription factor family are conserved regulators involved in a wide range of biological processes, including cellular differentiation, stress responses, programmed cell death, and autophagy in both vertebrates and invertebrates [1,2,3]. In insects, FoxO functions as a key downstream effector of insulin/insulin-like growth factor signaling (IIS), where it negatively regulates growth and contributes to the control of molting, metamorphosis, and developmental timing [4,5]. Ecdysteroid peaks can trigger metamorphosis in insects, and in honeybees, ecdysteroid peaks occur twice: at the prepupae 2 (PP2) and the pink- to red-eyed pupal stage (Pp) [6]. In insects, FoxO expression is closely associated with these hormonal peaks, suggesting a potential role in 20E-mediated developmental regulation [4,7]. Functional studies have shown that FoxO depletion in fifth-instar *Helicoverpa armigera* larvae disrupts molting progression and downregulates genes involved in 20E signaling [4]. In *Drosophila*, FoxO has also been reported to influence ecdysteroid production and critical weight (i.e., the minimum weight for pupation commitment) determination through interaction with Ultraspiracle (USP), further supporting its involvement in the ecdysone signaling network [8]. Previous studies reported that elevated *dFOXO* expression during early larval development inhibits larval growth, and its induction in third-instar larvae further results in decreased body size [9]. Additionally, disruption of FoxO activity has been shown to postpone adult emergence and reduce organismal size, likely due to its essential role in Insulin/insulin-like growth factor signaling (IIS)-dependent growth regulation [10].

The transition from larva to pupa during insect metamorphosis entails widespread breakdown of larval tissues and remodeling of pupal structures, including the midgut, fat body, and compound eyes in *Drosophila* [11,12]. Accumulating evidence indicates that FoxO also participates in lipid metabolic regulation across different animal species. In mammalian adipocytes, FoxO1 upregulates adipose triglyceride lipase (ATGL), driving triglyceride hydrolysis [13,14]. Concurrent knockdown of *Foxo1*, *Foxo3*, and *Foxo4* in mouse liver leads to lipid accumulation [15]. In insects, 20E-dependent activation of FoxO enhances lipase expression and promotes fat body lipolysis during molting and metamorphosis in *Drosophila melanogaster* and *Bombyx mori* [7,16]. Previous studies have suggested a close relationship between autophagic activity and intracellular lipid turnover. For instance, blocking autophagy in cultured hepatocytes via *Atg5* RNAi or treatment with 3-methyladenine (3-MA) increases TG storage within lipid droplets (LDs) [17]. Similarly, in *Drosophila*, autophagy is required for lipid mobilization, and disruption of autophagy-related genes results in lipid droplet accumulation [18]. Autophagy deficiency also impairs hepatic lipid utilization, reducing the efficiency of energy production from stored lipids [19].

In a previous study, we demonstrated that 20E signaling promotes autophagy-dependent lipid mobilization in the honeybee fat body during pupal development [20]. FoxO is a central regulator of insulin signaling and is closely associated with insect molting and metamorphosis. In *Bombyx mori* and *Helicoverpa armigera*, FoxO expression increases during molting and pupation, coinciding with peaks in 20E levels [4,17]. Knockdown of *FoxO* inhibits the synthesis of molting hormone in *Tribolium castaneum* larvae, impairs lipolysis in the fat body, and consequently results in delayed pupation and larval mortality [21]. However, the mechanism by which FoxO mediates 20E-induced pupation and fat body remodeling in worker bee larvae remains unclear. Furthermore, how FoxO modulates the crosstalk between fat body autophagy and lipolysis also remains poorly defined. Therefore, RNA interference was employed in this study to investigate the functional role of FoxO in regulating pupal development, autophagy, and lipid metabolism in worker honeybee larvae.

Honeybees are social insects with a unique metamorphosis process involving two ecdysteroid peaks and dramatic fat body remodeling. Although FoxO function has been extensively studied in model insects such as *Drosophila*, its role in honeybee development remains largely unexplored. Given the distinct endocrine regulation and social evolutionary context of honeybees, studying FoxO in this system may provide insights into species-specific adaptations of conserved FoxO signaling pathways. In this study, we characterized the developmental expression pattern of FoxO in *Apis mellifera* and investigated its functional roles using RNA interference.

## 2. Materials and Methods

### 2.1. In Vitro Rearing of Larvae

One-day-old *Apis mellifera* larvae were obtained from colonies maintained at the experimental apiary of Shandong Agricultural University (Tai’an, China). All procedures involving experimental animals were reviewed and approved by the Animal Ethics Committee of Shandong Agricultural University. Larvae were reared in vitro according to established protocols [22]. Larvae were reared individually in 48-well culture plates containing 160 μL of artificial diet (Table 1) and maintained at 33 °C with 55% relative humidity. The diet was replaced daily. Once larvae attained a pupation weight of 160–180 mg, feeding was stopped, and they were moved to 24-well plates lined with sterile paper to facilitate pupation. Mortality of larvae and pupae was recorded daily, and dead individuals were removed immediately. At the end of the rearing experiment, surviving pupae and newly emerged adults were recorded to determine pupation and adult emergence rates.

### 2.2. Injection of RNAi Knockdown into the Larvae

Gene-specific primers containing T7 promoter sequences at the 5′ ends (Table S1) were designed to amplify the FoxO fragment by PCR. The purified PCR product was subsequently used as a template for dsRNA synthesis using the RiboMaxTM T7 transcription kit (Promega, Madison, WI, USA) following the manufacturer’s instructions. As a negative control, dsRNA against the green fluorescent protein (GFP) gene (GenBank: U87974) was prepared similarly. For RNA interference, thirty 5-day-old worker bee larvae were injected twice with 10 μg dsRNA into the hemocoel at 24 h intervals. Untreated larvae and those injected with an equivalent amount of *dsGFP* served as controls. Samples were collected 24 h after the second injection, corresponding to the 7-day-old larval stage. Each experimental group included at least three biological replicates, with 16 larvae per replicate.

Larval and pupal development were monitored every 12 h after dsRNA injection. Individuals were considered dead when they exhibited no movement in response to gentle stimulation and showed characteristic signs of tissue melanization or decomposition. Mortality was recorded separately during the larval and pupal stages. The mortality rate was calculated as:

$$\mathrm{Mortality}\;\mathrm{rate}\;(\%)=\frac{Number\;of\;dead\;individuals}{Total\;number\;of\;individuals\;at\;the\;beginning\;of\;the\;experiment}\;\times \;100$$

Mortality in the *dsFoxO* group was compared with that of the *dsGFP* control group at the corresponding developmental stage.

### 2.3. Measurement of the 20E Titer and AP Activity

Hemolymph was diluted in pre-chilled PBS containing phenylthiourea. Hemolymph samples were collected and centrifuged at 12,000 rpm for 5 min at 4 °C. The resulting supernatant was carefully collected into a fresh tube, and a 50 μL aliquot was diluted 1:4 (*v*/*v*). The concentration of 20E was quantified using a commercial ELISA kit (Enzyme-linked Biotechnology, Shanghai, China) following the manufacturer’s protocol. All measurements were carried out in five independent biological replicates.

For biochemical assays, fat body tissues from three larvae were homogenized in phosphate-buffered saline (PBS) at a 1:9 (*w*/*v*) ratio for 180 s, followed by centrifugation at 12,000 rpm for 10 min at 4 °C. The supernatants were then used to measure TG content and the activities of lipase, acetyl-CoA carboxylase (ACC), fatty acid synthase (FAS), and acid phosphatase (AP) using commercial assay kits (Enzyme Linkage Biotechnology, Shanghai, China), following the manufacturer’s protocols. All measurements were carried out in five independent biological replicates.

### 2.4. Quantitative Real-Time PCR (qRT-PCR)

Total RNA isolation from fat body samples was performed with TRIZOL reagent (Takara Bio, Shiga, Japan) following the supplier’s instructions. 1 µg of total RNA was reverse-transcribed into cDNA. First-strand cDNA was synthesized from mRNA using Evo M-MLV Premix (Accurate Biotechnology, Changsha, China) following the provided instructions. Quantitative real-time PCR (qRT-PCR) was performed on an Applied Biosystems 7500 Real-Time PCR System (Waltham, MA, USA) using TransStart^®^ Tip Green qPCR SuperMix (TransGen Biotech, Beijing, China) in accordance with the supplier’s instructions. Gene-specific primers were designed and synthesized by Sangon Biotech (Shanghai, China), and their sequences are listed in Table S1. Each experimental group involved a minimum of five biological replicates and three technical replicates. *β-actin* gene (GenBank: XM_017065464) was used as the internal reference gene for normalization. Transcript abundance was calculated using the comparative 2^−ΔΔCt^ approach [23].

### 2.5. LysoTracker Red Staining and TEM Analysis

Larval fat body tissues were dissected into small fragments, washed with Ringer’s solution, and stained with LysoTracker Red (Invitrogen, Carlsbad, CA, USA) to assess autophagy using a Leica TCS SPE confocal microscope (Carl Zeiss, Oberkochen, Germany).

For ultrastructural analysis, tissues were fixed in 2.5% glutaraldehyde for 12 h at 4 °C, followed by post-fixation in 0.5% osmium tetroxide for 2 h. The tissues were then embedded in Epon resin and sectioned into ultrathin slices (60 nm). After staining with Reynolds’ lead citrate, sections were examined using a transmission electron microscope (H7800, Hitachi, Tokyo, Japan) to identify autophagosomes and autolysosomes. Autolysosomes contained residual organelle structures and partially degraded material, while autophagosomes (1–5 μm) enlarged progressively during pupation. Three biological replicates were performed, each pooling fat body tissue from 10 larvae, with all imaging conducted under identical conditions.

### 2.6. Staining of LDs

Fat body tissues were isolated from treated *Apis mellifera* larvae in bee Ringer’s solution and fixed with 4% paraformaldehyde at 4 °C for 12 h. Following fixation, tissues were embedded and sectioned into 7-μm transverse slices using a cryostat microtome. Lipid droplets were visualized by incubating the sections with Nile red solution (20 μg/mL) for 2 h at room temperature. After rinsing three times with phosphate-buffered saline (PBS), fluorescence signals were examined and imaged using a Leica TCS SPE confocal laser scanning microscope (Carl Zeiss, Oberkochen, Germany).

### 2.7. Histological Analysis

Fat body tissues were dissected and fixed in 4% paraformaldehyde overnight at 4 °C. Samples were dehydrated through a graded ethanol series, embedded in paraffin, sectioned at 5 μm thickness, stained with hematoxylin for 5 min and eosin for 2 min, and observed using an Olympus BX53 microscope (Olympus Corporation, New York, NY, USA).

### 2.8. Lipidomics Analysis

Lipidomic profiling was carried out by Wuhan Metware Biotechnology Co., Ltd. (Wuhan, China) using an ExionLC AD ultra-performance liquid chromatography system coupled with a QTRAP^®^ 6500+ mass spectrometer (Sciex, Framingham, MA, USA). Chromatographic separation was conducted using a Thermo Accucore C30 column (Thermo Fisher Scientific, Waltham, MA, USA) under gradient elution conditions. Mass spectrometric detection was performed using an electrospray ionization (ESI) source operating in both positive and negative ionization modes under Analyst 1.6.3 software control (Sciex). Instrument parameters were optimized according to standard operating procedures provided by the analytical platform. Lipid metabolites were detected using multiple reaction monitoring (MRM) mode with nitrogen as the collision gas. Instrument calibration and parameter optimization were carried out according to standard operating procedures provided by the manufacturer.

### 2.9. Statistical Analysis

Statistical analyses were conducted using SAS software version 9.1 (SAS Institute, Cary, NC, USA). Experimental data are expressed as the mean ± SEM from at least three independent biological replicates. Comparisons between two experimental groups were performed using Student’s *t*-test. Differences were considered statistically significant at *p* < 0.05 and highly significant at *p* < 0.01.

## 3. Results

### 3.1. Temporal and Spatial Expression Profiles of FoxO

**Figure 1:**
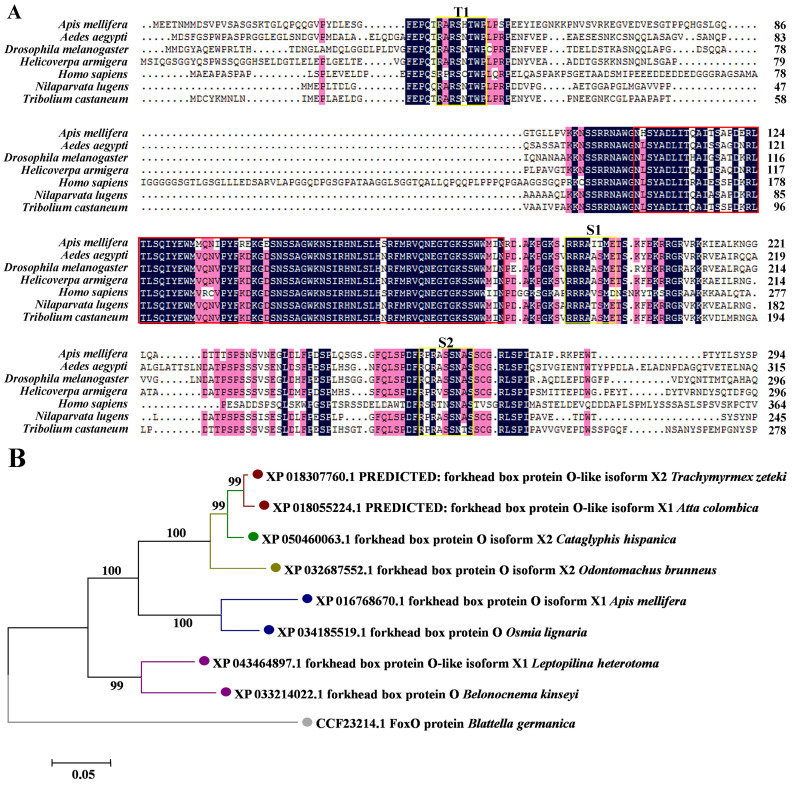
Bioinformatics analysis of FoxO in *Apis mellifera*. (**A**): Multiple sequence alignments of FoxO amino acids from seven insect species. The forkhead (FH) domain of FoxO is boxed in red. T1, S1 and S2 Akt target sequences are indicated in yellow. (**B**): Phylogenetic analyses of FoxO with FoxO sequences from 9 insect species. The scale bar indicates substitutions per position.

The honeybee FoxO gene sequence was obtained from the NCBI database (https://www.ncbi.nlm.nih.gov, accessed on 15 March 2025), and its structural characteristics were analyzed using online bioinformatics tools. The open reading frame (ORF) of FoxO is 1671 bp in length and encodes a protein of 556 amino acids with a predicted molecular weight of 60.8 kDa. Further analysis of the amino acid sequence of FoxO using National Center for Biotechnology Information (NCBI) tools revealed that it shares a typical conserved structural domain, the forkhead (FH) domain (94–183 amino acids), with its counterparts in other insects. Two potential Akt phosphorylation sites (Thr45 and Ser263) were identified in honeybee FoxO. Site T1 was located in Thr45 of FoxO and site S2 was located in Ser263, but site S1 was not found in FoxO of honeybees (Figure 1A). Two potential Akt phosphorylation sites in FoxO were consistent with the Akt consensus target sequence (RxRxxS/T). The Akt phosphorylation site is also conserved in the protein in terms of its relative position and sequence. To examine the evolutionary relationships of honeybee FoxO at the amino acid level with homologous FoxO sequences from other insect species, a phylogenetic tree was constructed using MEGA 7.0 software [24]. Full-length amino acid sequences were used for alignment and tree construction. Phylogenetic analysis of FoxO proteins from nine arthropod species showed that AmFoxO shares high sequence similarity with FoxO homologs from other insects, indicating that this gene is evolutionarily conserved and may have conserved functions across species. However, differences in phosphorylation sites (e.g., absence of S1 site in honeybee) and expression patterns may contribute to species-specific roles. AmFoxO showed the highest identity with FoxO of *Osmia lignaria* (74.4%) (Figure 1B).

**Figure 2:**
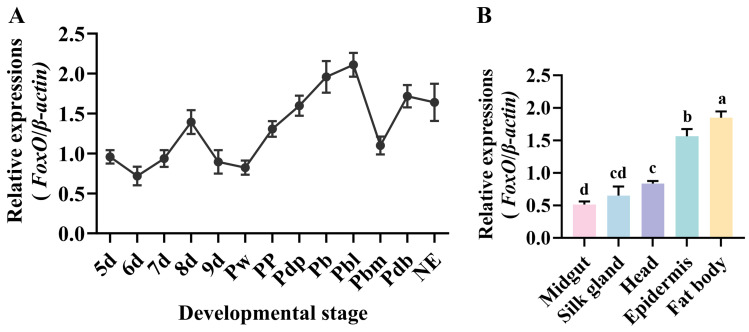
Expression patterns of *FoxO* in *Apis mellifera*. (**A**): qRT-PCR analysis of *FoxO* mRNA levels at different developmental stages. (**B**): Relative mRNA levels of *FoxO* in five tissues of 7-day-old larvae. Pw: white-eyed pupa; PP: pink-eyed/pharate adult transition; Pdp: dark pink-eyed pharate-adult; Pb: brown-eyed pharate adult with unpigmented cuticle; Pbl: brown-eyed pharate adult with lightly pigmented cuticle; Pbm: brown-eyed pharate adult with intermediate pigmentation; Pdb: brown-eyed pharate adult with dark pigmentation; NE: newly emerged adult. Data are expressed as mean ± SEM (*n* = 5). Different lowercase letters indicate significant differences among groups (*p* < 0.05).

To investigate the potential role of FoxO during honeybee development, its transcript abundance was examined across developmental stages. *FoxO* mRNA was detected throughout both larval and pupal development, and was higher in the prepupal stage compared to the larval stage in honeybees. During the pupal stage, *FoxO* mRNA expression gradually increased and reached a peak at the Pbl (brown-eyed pharate adult with lightly pigmented cuticle) stage (Figure 2A). Tissue-specific expression analysis in 7-day-old larvae revealed that *FoxO* was ubiquitously expressed in all examined tissues. Expression levels were lowest in the head, whereas the highest expression was observed in the fat body (Figure 2B), suggesting a potential role of FoxO in regulating energy metabolism and tissue remodeling in this organ.

### 3.2. FoxO Promotes Pupation

**Figure 3:**
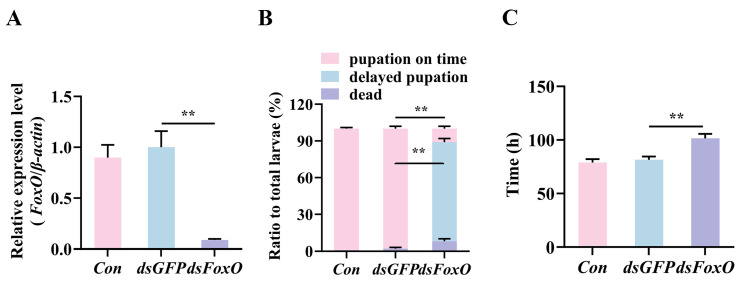
Knockdown of *FoxO* by dsRNA injection in 5-day-old larvae inhibits pupation. (**A**): qRT-PCR validation of RNA interference efficiency measured 24 h after dsRNA injection. (**B**,**C**): Statistical analysis of the time to pupation and the percentages of different phenotypic outcomes. Data are expressed as mean ± SEM (*n* = 5). ** *p* < 0.01.

Our previous work showed that 20E promotes pupation and inhibits the growth of honeybee larvae [25]. To investigate the role of FoxO in metamorphic development, FoxO expression was silenced by injecting dsRNA targeting FoxO into the hemocoel of 5-day-old larvae. qRT-PCR analysis confirmed that *FoxO* expression was significantly reduced in the *dsFoxO*-treated group compared with the *dsGFP* control (Figure 3A). In addition, larvae injected with *dsGFP* initiated pupation at 82 h post-injection, whereas the *dsFoxO*-injected larvae started to enter the pupal stage at 101 h after injection (Figure 3B). *FoxO* knockdown resulted in pronounced developmental defects, including delayed pupation, reduced survival (80% larval survival), low normal pupation rate (10%), and a high proportion of delayed or abnormal pupation (70%). In addition, most *dsFoxO*-treated individuals failed to complete metamorphosis and died during the pupal stage, exhibiting abnormal morphological phenotypes (Figure 3C).

### 3.3. FoxO Mediates Ecdysteroid Biosynthesis and 20E Signaling

**Figure 4:**
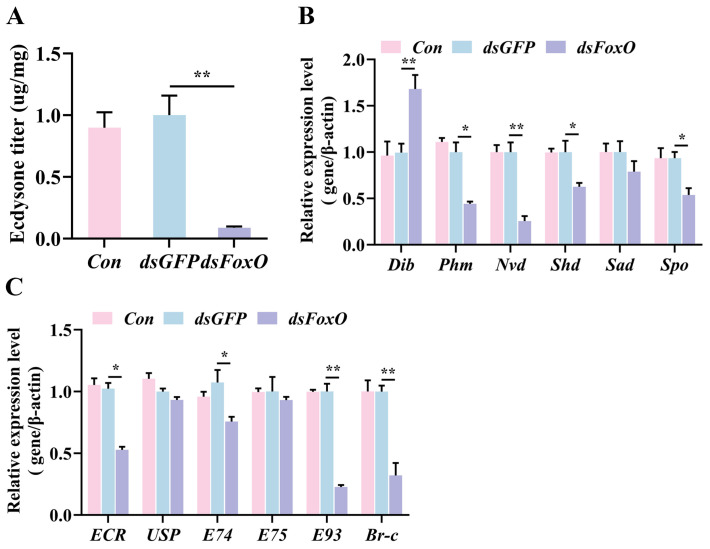
Effects of *dsFoxO* injection in 5-day-old larvae on ecdysteroid biosynthesis and 20E signaling in *Apis mellifera*. (**A**): 20E titer measured 24 h after *dsFoxO* treatment. (**B**,**C**): Relative expression levels of ecdysteroid synthesis genes and 20E signaling-related genes quantified by the 2^−ΔΔCT^ method. Larvae (6 days old) received 10 µg of dsRNA; untreated (Con) and *dsGFP*-injected larvae were used as controls. Data are expressed as mean ± SEM (*n* = 5). * *p* < 0.05, ** *p* < 0.01.

Compared with *dsGFP* injection, *dsFoxO* injection significantly reduced the 20E titer in larval hemolymph as well as the expression of genes involved in ecdysteroid biosynthesis (*dib*, *phm*, *nvd*, *sad*, *shd*, *spo*) at 24 h post-injection (Figure 4A,B). Additionally, the mRNA levels of key components in the 20E signaling pathway, including *ECR*, *E74*, *E93*, and *Br-c*, were significantly reduced in *dsFoxO*-injected larvae (Figure 4C). These data suggest that FoxO regulates the pupation time of larvae by modulating ecdysteroid biosynthesis and 20E signaling.

### 3.4. FoxO Promotes Lipid Droplet Breakdown

**Figure 5:**
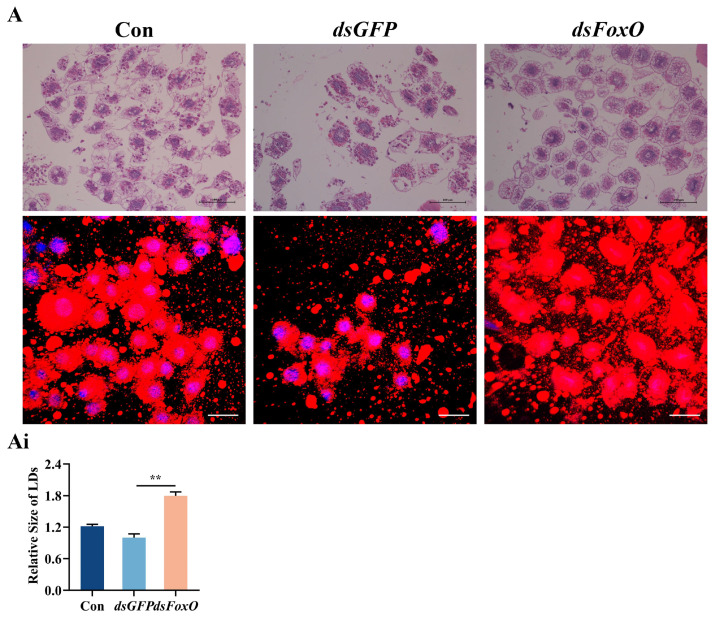
FoxO promotes LDs breakdown in the larval fat body. (**A**): Representative H&E and Nile Red staining images showing fat body structure. Lipid droplets were labeled with Nile Red (red), and nuclei were counterstained with DAPI (blue). Scale bar = 100 μm. (**Ai**) Quantification of LD size from (**A**). Data are expressed as mean ± SEM (*n* = 5). ** *p* < 0.01.

To examine the role of FoxO in fat body remodeling, histological analysis was performed 24 h after dsRNA injection. Hematoxylin and eosin (H&E) staining revealed that *FoxO* knockdown in 7-day-old larvae inhibited fat body dissociation. In addition, Nile Red staining showed a marked increase in both the size and number of lipid droplets (LDs) in *dsFoxO*-treated larvae compared with controls (Figure 5A,Ai).

### 3.5. FoxO Promotes Fat Body Lipolysis

**Figure 6:**
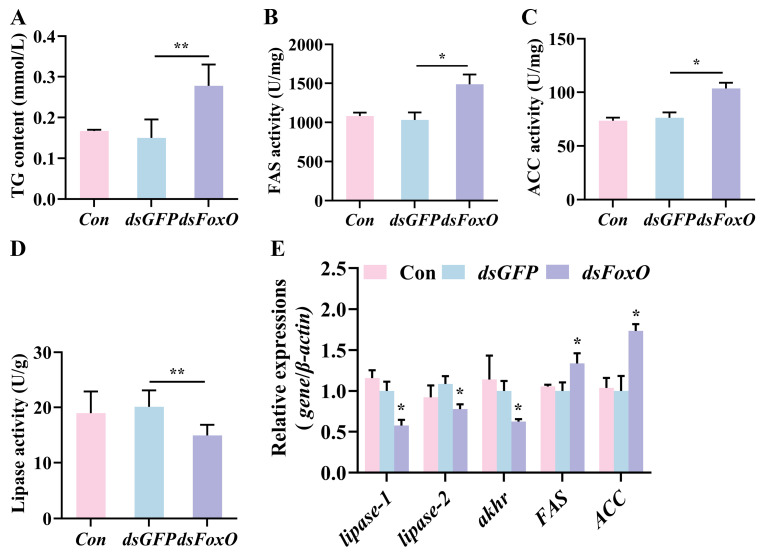
FoxO regulates lipid metabolism in larval fat bodies. (**A**–**D**) Triglyceride (TG) content (**A**), fatty acid synthase (FAS) activity (**B**), acetyl-CoA carboxylase (ACC) activity (**C**), and lipase activity (**D**) measured 24 h after dsRNA injection. (**E**): Relative transcript levels of *lipase-1*, *lipase-2*, *akhr*, *FAS*, and *ACC* determined by the 2^−ΔΔCT^ method. Data are expressed as mean ± SEM (*n* = 5). * *p* < 0.05, ** *p* < 0.01.

Quantitative analysis indicated that TG content in larval fat bodies was significantly higher 24 h after *dsFoxO* injection compared to the *dsGFP* injection (Figure 6A). Consistently, the activities of FAS and ACC were significantly elevated following *FoxO* knockdown (Figure 6B,C). Additionally, the knockdown of *FoxO* also significantly decreased lipase activity in larval fat body (Figure 6D). At the transcriptional level, FoxO silencing significantly downregulated lipolytic genes (*lipase-1* and *akhr*) while upregulating lipogenic genes (*FAS* and *ACC*) (Figure 6E).

### 3.6. Lipidomic Analysis of Worker Bee Larvae Following dsFoxO Injection

To further evaluate the impact of FoxO on lipid metabolism, lipidomic profiling was performed 24 h after dsFoxO injection. As no significant differences were detected between the Con and *dsGFP* groups, the *dsGFP* group was used as the control in the lipidomics analysis. Pearson correlation analysis was applied to assess the reproducibility among QC samples, with a stronger correlation of QC samples (|r| closer to 1) indicating high stability of the analytical process and reliable data quality. In this study, QC samples showed strong stability, with correlation coefficients exceeding 0.99 (Supplemental Figure S1). Additionally, the peaks in the TIC chromatograms of QC samples obtained in the positive and negative ion modes overlapped substantially (Supplemental Figure S2). These results confirmed the stability of the system for metabolomics analysis. Orthogonal projections to latent structures discriminant analysis (OPLS-DA) was used to evaluate group differences and identify variation in metabolite profiles. The *dsGFP* group (CK) and *dsFoxO* group were distinctly separated, revealing significant differences in lipid metabolites between samples (Supplemental Figure S3).

**Figure 7:**
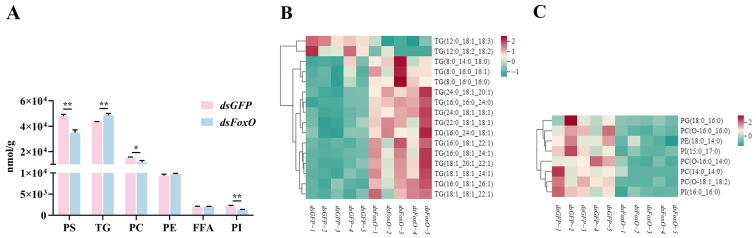
Lipid composition changes in *Apis mellifera* larvae following *dsFoxO* injection. (**A**): Overall lipid composition in *dsGFP*- and *dsFoxO*-injected larvae at 24 h post-injection. (**B**): Heatmap of significantly altered TG species. (**C**): Heatmap of significantly altered PC species. Data are expressed as mean ± SEM (*n* = 5). * *p* < 0.05, ** *p* < 0.01.

Compared with controls, *dsFoxO*-treated larvae exhibited significantly elevated TG levels but reduced phospholipid contents, including phosphatidylserine (PS), phosphatidylcholine (PC), and phosphatidylinositol (PI) (Figure 7A). Further lipid species analysis revealed that most differentially regulated TG species ranged from C44 to C60 and were significantly upregulated in the *dsFoxO* group (Figure 7B, Supplemental Figure S4A,B). In contrast, most altered PC species were significantly decreased (Figure 7C, Supplementary Figure S4C,D). These lipidomic alterations indicate that FoxO not only promotes TG breakdown but also modifies phospholipid composition, reflecting membrane remodeling and shifts in energy metabolism during the pupal stage.

**Figure 8:**
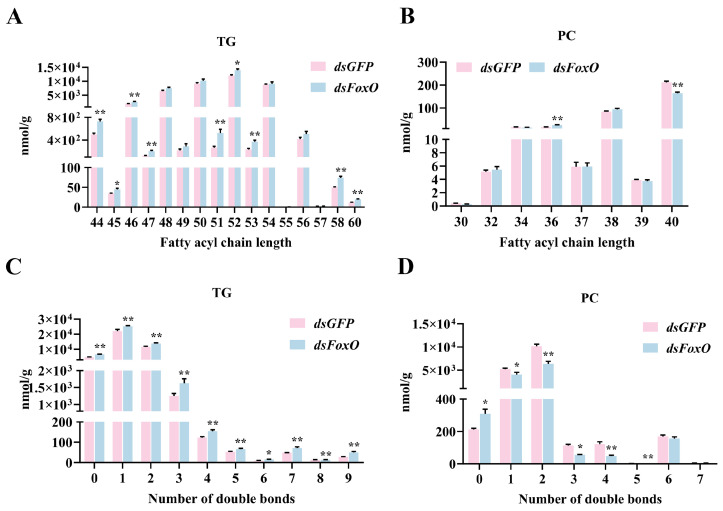
Alterations in fatty acyl chain characteristics of TG and PC in *Apis mellifera* larvae 24 h after *dsGFP* or *dsFoxO* injection. (**A**,**B**): Changes in acyl chain length of TG (**A**) and PC (**B**). (**C**,**D**): Variation in the number of double bonds in TG (**C**) and PC (**D**). Data are expressed as mean ± SEM (*n* = 5). * *p* < 0.05, ** *p* < 0.01.

Analysis of fatty acyl chain composition showed that *FoxO* knockdown significantly affected both chain length and unsaturation levels of lipids. Specifically, very-long-chain fatty acids (C ≥ 44) were markedly altered following *dsFoxO* treatment (Figure 8). Compared with controls, *dsFoxO* injection resulted in a significant reduction in TG species with 44–60 carbon atoms and PC species with 36–40 carbon atoms (Figure 8A,B). In addition, the degree of unsaturation was significantly decreased, as reflected by reduced abundance of TG species containing 0–9 double bonds and PC species containing 0–7 double bonds (Figure 8C,D). These results demonstrate that FoxO regulates lipid remodeling during pupation by modulating fatty acid chain length and saturation in both storage and membrane lipids.

**Figure 9:**
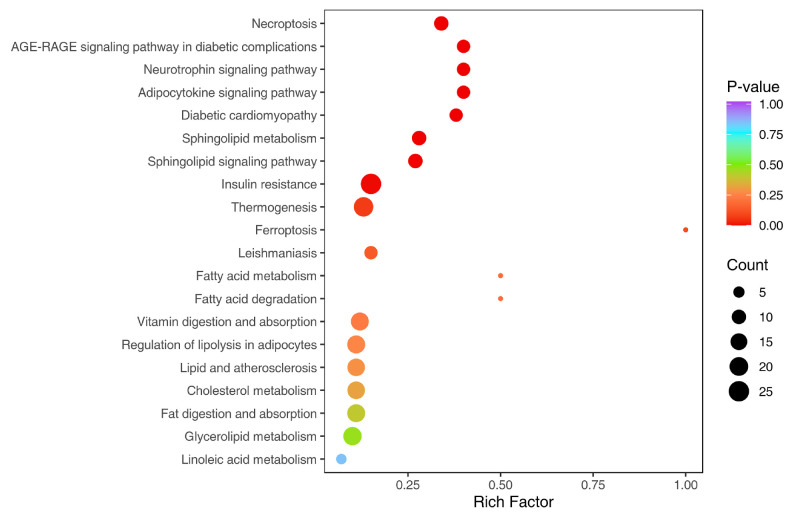
KEGG pathway enrichment analysis of differentially abundant lipids between *dsGFP*- and *dsFoxO*-injected larvae (24 h post-injection).

Furthermore, Kyoto Encyclopedia of Genes and Genomes (KEGG) pathway enrichment analysis was conducted to identify the biological functions associated with differential lipids. The results showed that lipids altered between the *dsGFP* and *dsFoxO* groups were significantly enriched in pathways including necrosis, adipocytokine signaling pathway, neurotrophin signaling pathway, SP metabolism, insulin resistance, and lipid and atherosclerosis (Figure 9).

### 3.7. FoxO Promotes Fat Body Autophagy

**Figure 10:**
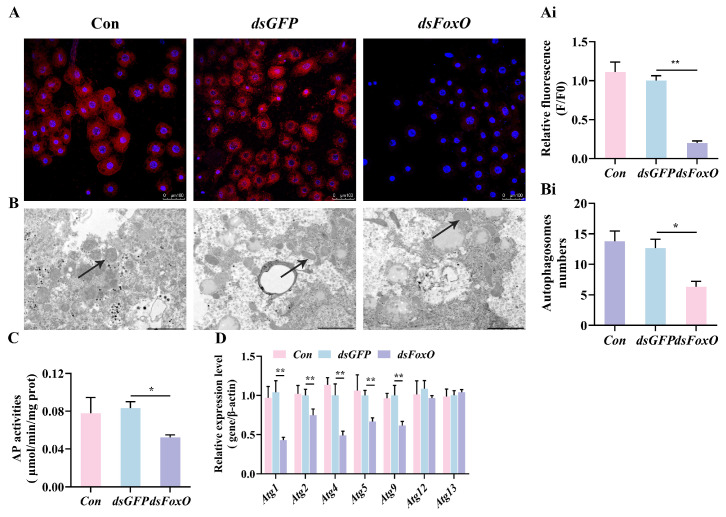
Reduction in autophagy by injection of *dsFoxO* into 5-day-old larvae. (**A**,**Ai**) LysoTracker Red staining of fat bodies 24 h after *dsFoxO* injection; quantification in (**Ai**). Red, LysoTracker; blue, DAPI-stained nuclei. Scale bar, 100 μm. (**B**,**Bi**) TEM images showing autophagosomes (arrows); quantification in (**Bi**). Scale bar, 20 μm. (**C**) AP activity in larval fat bodies. (**D**) Relative transcript levels of *Atg* genes. Data are expressed as mean ± SEM (*n* = 5). * *p* < 0.05, ** *p* < 0.01.

LysoTracker Red fluorescence showed a marked decrease in fluorescence intensity in the fat body 24 h after *dsFoxO* injection compared with the *dsGFP* control (Figure 10A,Ai). TEM revealed that autophagosomes were rarely observed in *dsFoxO*-treated larvae, whereas they were readily detectable in the *dsGFP* group (Figure 10B,Bi). In addition, AP activity was significantly reduced following *FoxO* silencing (Figure 10C). qRT-PCR analysis indicated that the expression levels of six *Atg* genes were significantly decreased 24 h after *dsFoxO* injection (Figure 10D). Collectively, these results suggest that *FoxO* knockdown suppresses autophagic processes during larval fat body remodeling in honeybees.

## 4. Discussion

### 4.1. FoxO Is Involved in Regulating Larval Pupation

To investigate the functional role of FoxO during the larval–pupal transition, RNA interference was used to silence FoxO expression in six-day-old honeybee larvae. FoxO knockdown resulted in significant developmental delays, abnormal pupal morphology, and high pupal mortality. Similar phenotypes have been reported in other model organisms, including *Caenorhabditis elegans*, where FoxO disruption delays molting progression [26], and *Helicoverpa armigera*, where FoxO silencing leads to delayed pupation and abnormal metamorphosis [4]. In *Drosophila*, FoxO mutants exhibit developmental retardation across multiple life stages [10], further supporting a conserved role of FoxO in developmental timing control. We hypothesized that the delayed pupation observed in *dsFoxO*-injected larvae may result from disruption of ecdysone biosynthesis and 20E signaling. To further support this hypothesis, we measured hemolymph 20E titers and the expression of genes involved in ecdysteroid synthesis following *FoxO* knockdown. Both 20E levels and the transcript abundance of several ecdysteroid biosynthesis-related genes were significantly decreased 24 h after *dsFoxO* injection. Additionally, the transcript levels of several key genes of the 20E signaling pathway were significantly reduced in *dsFoxO*-injected larvae. Taken together, these results indicate that FoxO participates in the regulation of both ecdysteroid biosynthesis and 20E signaling.

### 4.2. FoxO Coordinates Lipid Mobilization During Metamorphosis

Fat body remodeling is a hallmark of insect metamorphosis, ensuring sufficient energy supply for pupal development. Our previous study demonstrated that 20E induces autophagy-dependent lipid mobilization in the honeybee fat body [21]. FoxO has also been implicated in the regulation of both ecdysteroid biosynthesis and 20E signaling. In the present study, FoxO knockdown resulted in increased triglyceride accumulation, reduced lipase activity, and downregulation of lipolytic genes (*lipase-1* and *akhr*), indicating impaired lipid mobilization. These findings are consistent with studies in other insects, where FoxO promotes lipolysis by regulating lipase gene expression. In *Bombyx mori*, FoxO enhances lipid breakdown by activating lipolytic pathways [7], while in *Drosophila*, FoxO activation upregulates *brummer* expression and promotes triglyceride mobilization [27]. In mammals, FoxO1 overexpression similarly modulates lipid metabolism in the liver [28]. Collectively, these studies support a conserved function of FoxO in coordinating lipid mobilization during insect development. Interestingly, lipidomic analysis in this study revealed that FoxO not only regulates triglyceride breakdown but also remodels phospholipid composition, indicating that FoxO contributes to both energy supply and membrane remodeling required for tissue differentiation. The present study suggests that FoxO knockdown leads to a marked reduction in 20E levels, which may contribute to the observed developmental and metabolic phenotypes. However, whether FoxO directly regulates lipid metabolism or acts primarily through 20E-mediated pathways remains to be elucidated.

### 4.3. FoxO Mediates Autophagy During Fat Body Remodeling

Most larval tissues undergo extensive remodeling or degradation to form adult structures through tightly coordinated processes involving cell proliferation, differentiation, and programmed cell death (PCD) [29]. Autophagy has been demonstrated to contribute to PCD in insects [30]. FoxO is involved in autophagy in mammals and *Drosophila* [31,32]. In *Helicoverpa armigera*, FoxO overexpression promotes autophagy and suppresses cell proliferation [4]. In this study, *FoxO* knockdown markedly reduced LysoTracker fluorescence intensity, decreased autophagosome formation, and downregulated multiple *Atg* genes in the fat body. These results indicate that FoxO is required for the activation of autophagy during the transition from larval to pupal stages in *Apis mellifera*. Moreover, our lipidomics clearly demonstrate that FoxO is associated with lipid metabolism in worker bee larvae.

In somatic cells, autophagy is essential for maintaining lipid homeostasis, and suppression of autophagy-related genes promotes lipid droplet accumulation [19]. In mammals, impaired autophagy compromises hepatic lipid utilization, thereby reducing the efficiency of lipid-derived energy production [33]. These findings support a conserved relationship between autophagy and lipid metabolism across biological systems, including insects such as *Apis mellifera* [18,34]. Comparable observations have also been reported in fungi, where loss of autophagy genes in *Metarhizium robertsii* (Hypocreales: Clavicipitaceae) inhibits lipid droplet degradation under nutrient-limited conditions [20]. Consistent with these findings, our results show that *FoxO* knockdown suppresses autophagy, leading to lipid droplet accumulation and impaired lipid mobilization. Thus, FoxO functions as a central regulatory hub linking autophagy and lipid catabolism, ensuring sufficient energy supply during pupal development.

We demonstrate that FoxO is essential for normal pupation, acting through the regulation of ecdysteroid biosynthesis, autophagy, and lipolysis in the fat body. Our findings confirm the presence of a FoxO-autophagy-lipolysis axis in honeybees, providing new insights into the conserved mechanisms linking endocrine signaling, energy metabolism, and developmental transitions in insects.

## 5. Conclusions

**Figure 11:**
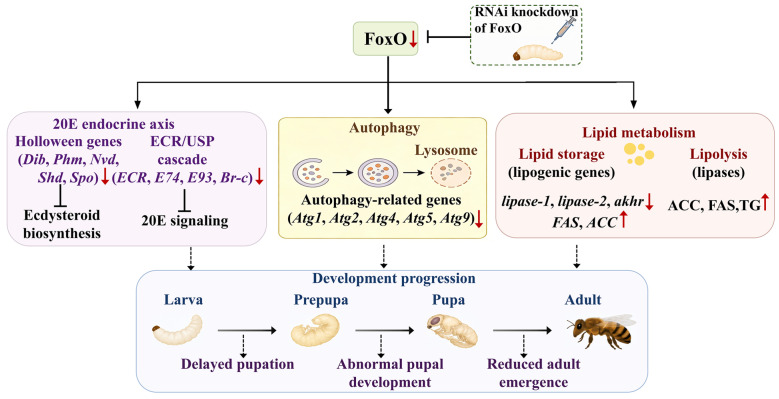
Proposed model of FoxO-mediated regulation of ecdysteroid signaling, autophagy, and lipid metabolism during honey bee larval development. FoxO knockdown affects 20E endocrine axis and metabolic processes. Solid arrows indicate experimentally supported regulatory relationships, whereas dotted arrows represent putative or indirect interactions based on current evidence. The interaction between autophagy and lipid metabolism is considered a potential crosstalk that has not been directly demonstrated in this study. Developmental stages are reorganized as a continuous larva–prepupa–pupa–adult timeline for clarity.

In this study, we systematically characterized the structural features, evolutionary conservation, and developmental expression of the honeybee FoxO gene, and revealed its indispensable role in larval–pupal metamorphosis. Functional analyses revealed that FoxO regulates pupation by modulating ecdysteroid biosynthesis and 20E signaling, thereby ensuring proper developmental timing. Moreover, FoxO coordinates energy metabolism with morphogenesis by activating autophagy and promoting fat body lipolysis, thereby mobilizing stored lipids to fuel pupal development (Figure 11).

This study first elucidates the core regulatory function of the FoxO-autophagy-lipolysis axis in metamorphosis within honeybees. Beyond providing mechanistic insights into honeybee development, our work highlights the evolutionary conservation of FoxO function across taxa and opens new avenues for investigating how transcriptional networks couple metabolism to developmental plasticity. Such knowledge not only deepens our understanding of insect physiology but may also inform strategies for improving honeybee health and resilience in the face of environmental challenges.

## Figures and Tables

**Table 1:** Composition of larvae diets.

Ingredients	Content (%)
Royal jelly	50.00
Glucose	6.00
Fructose	6.00
Yeast extract	1.00
Sterile water	37.00
Total	100.00

## Data Availability

The raw data supporting the conclusions of this article will be made available by the authors on request.

## Supplementary Materials

The following supporting information can be downloaded at: https://www.mdpi.com/article/10.3390/insects17070684/s1. Figure S1: The correlation analysis of QC samples or honeybee larvae samples carried out in the present method; Figure S2: Total ions current (TIC) overlapping map of QC samples mass spectrometry results; Figure S3: The score plots of OPLS-DA pairwise comparisons of differential lipid metabolites; Figure S4: The lipid composition of *A. mellifera* larvae changed after injection of *dsFoxO*; Table S1: The primer sequences used for qPCR and dsRNA.
