# Supplementary material for: A FoxO–Autophagy–Lipid Mobilization Axis Regulates Fat Body Remodeling During Honeybee Metamorphosis
Source: Insects. 2026 Jul 1;17(7):684. doi: 10.3390/insects17070684 (PMC13410173; doi:10.3390/insects17070684)
Supplement: Supplementary file 1 [file insects-17-00684-s001.zip › Supplementary.pdf]

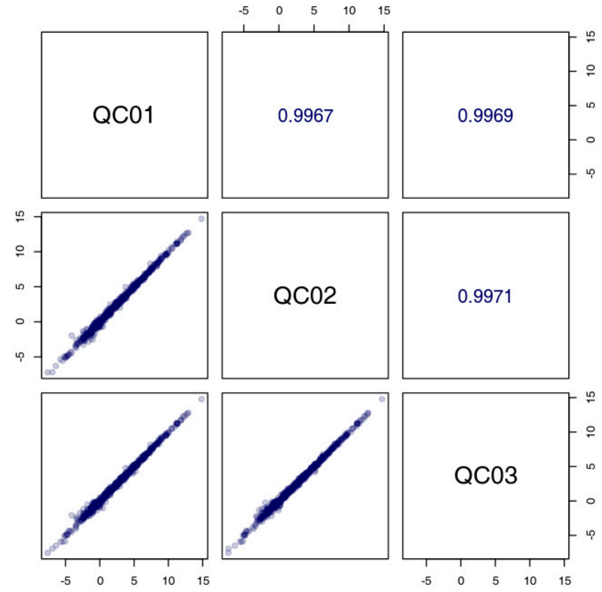

Fig. S1 The correlation analysis of QC samples or honeybee larvae samples carried out in the present method.

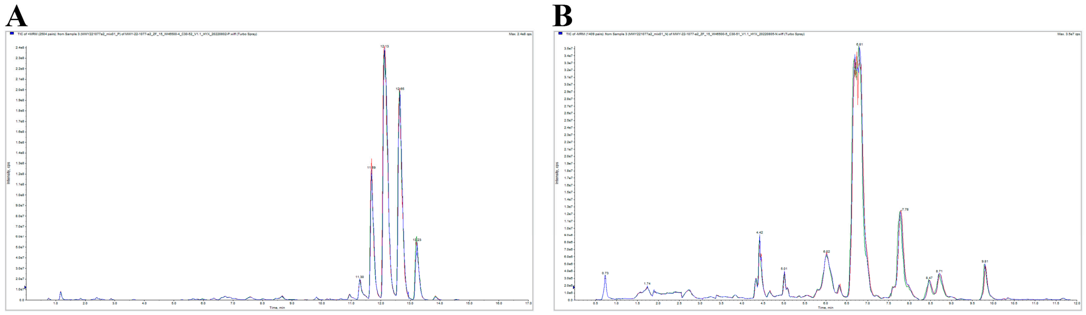

Fig. S2 Total ions current (TIC) overlapping map of QC samples mass spectrometry results. (A) in positive ion mode, (B) in negative ion mode.The high overlap of total ion current curves in lipid detection, characterized by consistent retention times and peak intensities, indicates excellent signal stability when analyzing the same sample at different times. The instrument's high stability provides crucial assurance for data reproducibility and reliability. Here, N denotes negative ion mode, while P denotes positive ion mode.

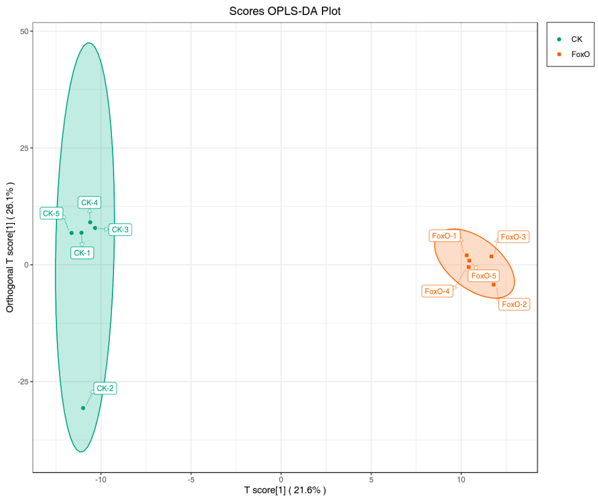

Fig. S3. The score plots of OPLS-DA pairwise comparisons of differential lipid metabolites. CK vs FoxO, 6-day-old larvae were injected with *dsFoxO*, with *dsGFP* (CK).

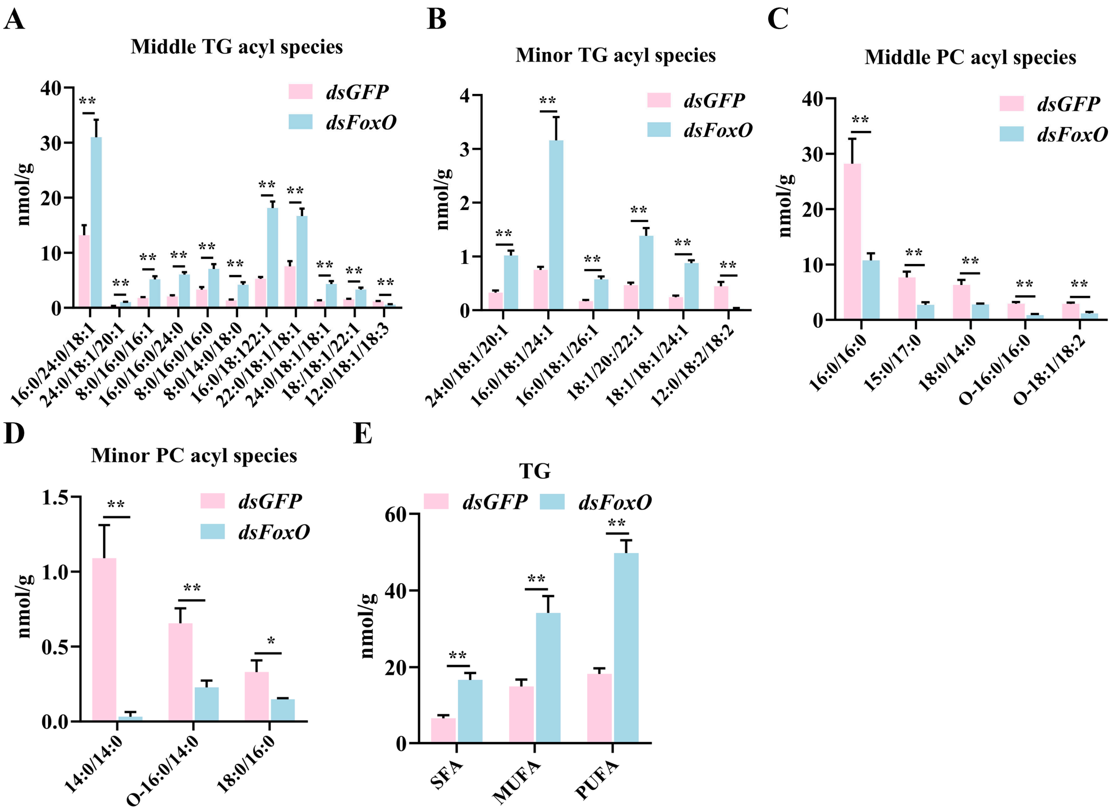

Fig. S4. The lipid composition of *A. mellifera* larvae changed after injection of *dsFoxO*. A-B: The middle and minor TG acyl species that showed significant changes in *A. mellifera* larvae injected of *dsGFP* (CK) and *dsFoxO* for 24 h. C-D: The middle and minor PC acyl species that showed significant changes in *A. mellifera* larvae injected of *dsGFP* (CK) and *dsFoxO* for 24 h. E: Changes in the contents of saturated fatty acids (SFA), monounsaturated fatty acids (MUFA), and polyunsaturated fatty acids (PUFA) in *A. mellifera* larvae injected of

dsGFP (CK) and *dsFoxO* for 24 h. Asterisks above the bars indicate significant differences between larvae injected of *dsGFP* (CK) and *dsFoxO* by using the student's *t*-test (*, $P<0.05$; **, $P<0.01$). Error bars indicate the mean±SEM of five independent biological experiments.
